# Association between malaria control and paediatric blood transfusions in rural Zambia: an interrupted time-series analysis

**DOI:** 10.1186/1475-2875-13-383

**Published:** 2014-09-26

**Authors:** Alison B Comfort, Janneke H van Dijk, Sungano Mharakurwa, Kathryn Stillman, Benjamin Johns, Payal Hathi, Sonali Korde, Allen S Craig, Nancy Nachbar, Yann Derriennic, Rose Gabert, Philip E Thuma

**Affiliations:** International Health Division, Abt Associates, Cambridge, MA USA; Clinical Research Department, Macha Research Trust, Choma, Zambia; Johns Hopkins Malaria Research Institute, Malaria Research Department, Macha Research Trust, Choma, Zambia; President’s Malaria Initiative, Bureau of Global Health, USAID, Washington District of Columbia, Washington, DC USA; President’s Malaria Initiative, Division of Parasitic Diseases and Malaria, Center for Global Health, Centers for Disease Control and Prevention, Lusaka, Zambia; Global Immunization Division, Center for Global Health, Centers for Disease Control and Prevention, Atlanta, GA USA; Macha Research Trust, Choma, Zambia; The Institute for Health Metrics and Evaluation, University of Washington, Seattle, WA USA

**Keywords:** Malaria control, Blood transfusion, Severe malarial anaemia, Paediatric hospital admissions, Zambia, Sub-Saharan Africa, Time series

## Abstract

**Background:**

Blood transfusions can reduce mortality among children with severe malarial anaemia, but there is limited evidence quantifying the relationship between paediatric malaria and blood transfusions. This study explores the extent to which the use of paediatric blood transfusions is affected by the number of paediatric malaria visits and admissions. It assesses whether the scale-up of malaria control interventions in a facility catchment area explains the use of paediatric blood transfusions.

**Methods:**

The study was conducted at a referral hospital for 13 rural health centres in rural Zambia. Data were used from facility and patient records covering all paediatric malaria admissions from 2000 to 2008. An interrupted time series analysis using an autoregression-moving-average model was conducted to assess the relationship between paediatric malaria outpatient visits and admissions and the use of paediatric blood transfusions. Further investigation explored whether the use of paediatric blood transfusions over time was consistent with the roll out of malaria control interventions in the hospital catchment area.

**Results:**

For each additional paediatric malaria outpatient visit, there were 0.07 additional paediatric blood transfusions (95% CI 0.01-0.13; *p* < 0.05). For each additional paediatric admission for severe malarial anaemia, there were 1.09 additional paediatric blood transfusions (95% CI 0.95-1.23; *p* < 0.01). There were 19.1 fewer paediatric blood transfusions per month during the 2004–2006 malaria control period (95% CI 12.1-26.0; *p* < 0.01), a 50% reduction compared to the preceding period when malaria control was relatively limited. During the 2007–2008 malaria control period, there were 27.5 fewer paediatric blood transfusions per month (95% CI 14.6-40.3; *p* < 0.01), representing a 72% decline compared to the period with limited malaria control.

**Conclusions:**

Paediatric admissions for severe malarial anaemia largely explain total use of paediatric blood transfusions. The reduction in paediatric blood transfusions is consistent with the timing of the malaria control interventions. Malaria control seems to influence the use of paediatric blood transfusions by reducing the number of paediatric admissions for severe malarial anaemia. Reduced use of blood transfusions could benefit other areas of the health system through greater blood availability, particularly where supply is limited.

## Background

Malaria is one of the leading causes of child mortality in sub-Saharan Africa (SSA). According to the most recent estimates from the World Health Organization World Malaria Report 2013, malaria accounted for approximately 627,000 deaths world-wide in 2012, of which almost 77% were among children under five years of age [1]. Malaria has been recognized as a major contributor to anaemia among children [2]. Severe malarial anaemia is a common presentation of severe malaria in children under five years of age [3], and is one of the primary causes of hospitalization and death for children in SSA [4]. Severe malarial anaemia was found in 17% of all malaria admissions among children aged zero to nine years in a study of 13 hospitals across Africa [5]. A review of studies on malaria control interventions in SSA found that moderate-to-severe anaemia in children is more than halved with the introduction of interventions, including insecticide-treated nets and intermittent presumptive treatment [6]. Most severe forms of malaria and most malaria-related deaths are caused by the parasite *Plasmodium falciparum* [7].

Evidence demonstrates that blood transfusions can save the lives of children with severe malarial anaemia [8, 9], and also shorten recovery time from anaemia [10]. Delays in the receipt of blood transfusions can significantly increase mortality among patients, particularly for those with severe malarial anaemia, and improved availability of blood could prevent some of these deaths [8, 11–14]. Specifically in SSA, evidence shows that anaemia as a result of malaria accounts for up to 70% of all paediatric blood transfusions [9]. Over 60% of deaths in children with severe malarial anaemia occur before a transfusion is given [15]. In Tanzania, malarial anaemia accounted for 98% of all paediatric blood transfusions among children receiving blood transfusions in the paediatric wards of two hospitals [11]. In Kenya, 88% of one hospital’s paediatric blood transfusions over the course of a year were for children with severe anaemia [12], likely associated with malaria.

Other frequent uses of blood transfusions, particularly in low- and middle-income countries, include severe obstetrical haemorrhage, trauma, and severe childhood anaemia, which is often but not always due to malaria [13, 16]. Despite the widespread need for blood transfusions for anaemia and other conditions, it is well documented that maintaining an adequate safe blood supply can be a significant challenge, particularly in SSA [13, 17, 18]. Efforts to maintain adequate blood supplies have become more challenging in recent years due to increasingly stringent blood safety and quality assurance practices to address risks related to infectious diseases (such as HIV, hepatitis B and C, and syphilis) and a related trend towards centralizing blood collection and management. Together, these factors can contribute to delaying the receipt of potentially life-saving blood [19, 20].

Given the extensive use of blood transfusions to treat severe malarial anaemia, particularly among children, reducing the incidence of malaria through improved malaria control could substantially reduce the need for paediatric blood transfusions. In turn, reductions in the use of paediatric blood transfusions could benefit non-malaria patients, such as trauma patients or patients with post-partum haemorrhage, particularly in areas with blood supply shortages. There is limited research assessing the effect of malaria control on the use of paediatric blood transfusions. Two studies focused on the effects of improved malaria treatment. In Zanzibar, the number of blood transfusions for children under five years of age fell by 67% with the introduction of artemisinin-based combination therapy (ACT) [21]. In Zambia, uncomplicated malaria patients receiving the combination therapy atovaquone-proguanil (AP) required no blood transfusions, while 8% of patients receiving sulphadoxine-pyrimethamine (SP) required a blood transfusion within 14 days of treatment initiation [22]. Only one study, conducted in Ghana, has focused on malaria control programmes more broadly; this study found that the frequency of blood transfusions for patients with confirmed malaria decreased from 24.1% to 11.3% following the implementation of Ghana’s malaria control program in one of Ghana’s districts [9].

Using retrospective time-series data from 2000 to 2008 from one hospital in Zambia, this study quantifies the extent to which the use of paediatric blood transfusions is associated with the number of paediatric malaria outpatient visits, paediatric malaria admissions, and paediatric admissions for severe malarial anaemia. Paediatric admissions for severe malarial anaemia are a sub-category of all paediatric malaria admissions; other malaria admissions include complications such as malarial anaemia, cerebral malaria, and the combination of these. This study tests the underlying assumption that a reduction in malaria outpatient visits is associated with a reduction in malaria admissions, which in turn may influence blood transfusion use. The rate at which malaria outpatient visits decrease over time relative to inpatient malaria admissions may depend on factors such as hospitals continuing to admit the most serious malaria cases. This study seeks to test these various relationships to better understand their associations with each other and, ultimately, their relationship with paediatric blood transfusion use.

The study then investigates whether the use of paediatric blood transfusions is associated with the scale-up of malaria control efforts in the hospital’s catchment area over time. Specifically, it assesses whether there is a difference in the rate of monthly use of paediatric blood transfusions before and after malaria control scale-up, as well as a difference in the average monthly use before and after scale-up. Not only does this study complement the evidence from Ghana on the effect of malaria control scale-up on paediatric blood use, it also quantifies the extent to which the use of paediatric blood transfusions varies with paediatric malaria visits and admissions.

This study also explores the seasonality of paediatric blood use, for which there is limited evidence. Countries with high seasonality of malaria incidence are those where 60% of the annual rainfall occurs within three months; these countries mostly include SSA countries located in the Sahel and sub-Sahel [23]. In Zambia, the number of paediatric malaria admissions peak following the peak in rainfall during the rainy season [24]. If paediatric blood use is seasonal, there may be implications for blood availability for non-malaria patients, particularly during the high malaria transmission months. The findings would not only substantiate the relationship between paediatric malaria visits and the use of paediatric blood transfusions, but also suggest that there may be potential benefits to non-malaria patients from reduced paediatric blood transfusions, particularly during the high malaria transmission season.

Finally, this study conducts exploratory analyses to identify whether total blood use at the hospital is influenced by blood use in the paediatric ward. It also tests whether use of blood transfusions in the paediatric ward affects use of blood transfusions in other patient wards, to identify potential spillover effects (or indirect benefits) for other patients. There are no known studies assessing the potential benefits of malaria control scale-up for non-malaria patients, by freeing up blood supply for other conditions.

## Methods

### Study area

This study was conducted in Zambia, a country in southern Africa with historically high prevalence of malaria that has made significant progress in reducing malaria morbidity and mortality over the past decade [25]. Zambia was an early adopter of effective malaria control interventions, including ACT as a first-line treatment, free rapid diagnostic tests (RDTs), mass distribution of insecticide-treated nets (ITNs), and wide deployment of targeted indoor residual spraying (IRS) [25]. The data for this study were collected from Macha Mission Hospital (MMH), a 208-bed facility (including 55 paediatric beds) located in rural Choma District in Southern Province. MMH serves as the referral hospital for 13 rural health centres in parts of Choma, Namwala and Kalomo Districts. The hospital has its own hospital-affiliated health centre, serving as an outpatient department. MMH has been involved in malaria research for over 20 years, and is currently the site for the Macha Research Trust, the successor to the Malaria Institute at Macha (MIAM) established in 2003. Macha was selected for this study given its extensive record-keeping during the last decade resulting in high-quality data on both malaria services and blood transfusions specifically.

Malaria transmission in MMH’s catchment area, covering approximately 160,000 people, has traditionally been hyperendemic transmission of *Plasmodium falciparum* [26]. *Anopheles arabiensis* are the main vector responsible for malaria transmission in the Macha area [27]. A 2002 entomological study conducted in the catchment area found that the entomological inoculation rate (EIR) was estimated to be 81 infected bites per person per year (unpublished observations; Siachinji *et al*. 2003); this EIR is similar to other countries in sub-Saharan Africa and lower than many countries in West Africa [28].

In Southern Province, there is one rainy season from November to April, followed by a cool dry season (April-August) and a hot dry season (August-November) [24]. Data show that paediatric malaria admissions at MMH closely follow the timing of the rainy season. During a severe drought in Southern Province from November 2004 to April 2005, malaria transmission was nearly zero resulting in very low paediatric malaria admissions. Normal rain returned during 2005–2006 rainy season, and paediatric malaria admissions increased, though they were lower than pre-drought admissions [24].

### Timeline of malaria control scale-up in MMH catchment area

**Figure 1:**
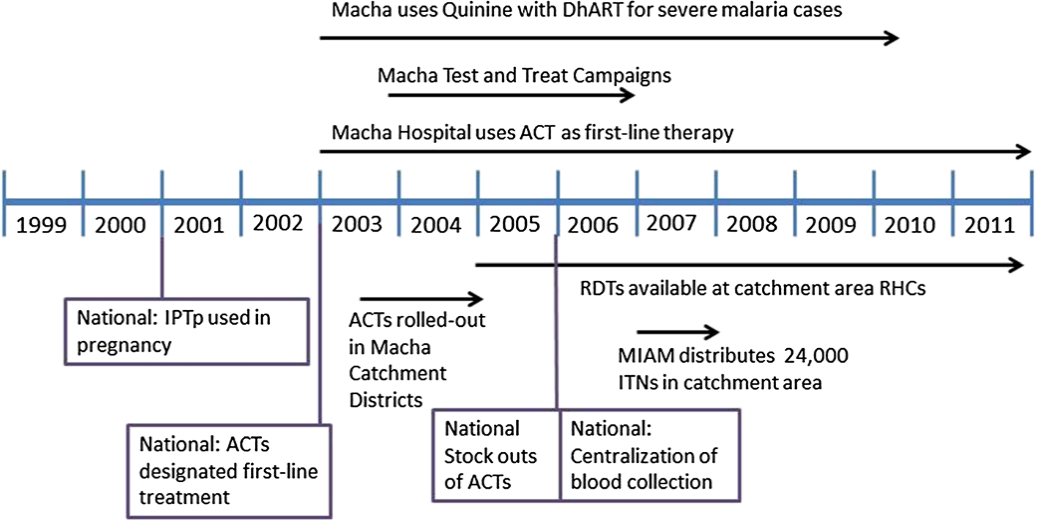
**Macha Mission Hospital malaria control interventions over time.** Diagram depicting both national and local changes in malaria control and blood transfusion policy.

In 2003, the Zambian government introduced a revised malaria treatment policy by including ACT, specifically artemether-lumefantrine (AL), making Zambia the first country in Africa to adopt ACT as the national first-line therapy for the treatment of uncomplicated malaria. MMH introduced ACT as its first line of treatment soon after the policy shift (Figure 1). Kalomo District, which includes part of MMH’s catchment area, was among the first seven districts in Zambia to receive ACT in early 2003 [29]; ACT then became available in Choma District in late 2003 and in Namwala District in late 2004. In late 2003, as part of a larger epidemiologic study, a community malaria test-and-treat and education campaign was carried out in a random sample of villages in the hospital catchment area. All consenting residents were screened for malaria by RDT, and those that tested positive were treated with ACT whether they were symptomatic or asymptomatic.

In 2005, the three districts in MMH’s catchment area received RDTs in line with the National Malaria Control Programme’s new policy to strengthen malaria diagnosis with RDTs [30]. In addition, ITN distribution was scaled up in Choma District from 2003 to 2005. In 2006, government purchasing challenges at the central level led to ACT shortages throughout Zambia; however, ACT stocks returned to adequate levels in 2007 (Thuma, unpublished data). In 2006, 1,000 ITNs were distributed in one community in MMH’s catchment area, but there was no widespread distribution of ITNs in other parts of the catchment area before 2007 [24]. While the Malaria Indicator Survey data are not disaggregated at the district level, province level data show that ITN ownership increased from 47% in 2006 to 70% in 2008 in Southern Province, but use of ITNs increased only marginally (from 30% to 32% of children under five) [31, 32]. In 2007, MIAM participated in the Ministry of Health’s ITN distribution campaign for the northern part of Choma District, distributing 24,000 ITNs through eight rural health centres surrounding the hospital; this effort raised the self-reported rate of ITN use in the previous night to over 80% in MMH’s catchment area [28]. Although targeted IRS was rolled out in Choma District in 2008, it was restricted to peri-urban areas, thereby not reaching MMH’s catchment area.

### Data sources

The study uses retrospective monthly time-series data, drawn from facility and patient records from 2000 to 2008. Inpatient malaria admission data, available from 2003 to 2008, were extracted from an electronic database that included all malaria admissions for patients five years and under. These data include patient age, sex, length of stay, malaria microscopy results, primary and secondary discharge diagnosis, treatment for malaria (including blood transfusions), and co-morbidities. Data on monthly under-five outpatient visits for malaria were extracted from the hospital-affiliated health centre disease aggregation forms and were available for the period 2000–2008.

Monthly blood transfusion data for the paediatric (five and under), maternity, women’s, men’s, and tuberculosis wards were collected from an electronic database tracking all blood transfusions given at MMH from 2000 through 2008. The database was periodically consolidated by Macha Research Trust using the hospital-based laboratory blood bank transfusion log books; the senior author on this study coordinated and supervised the quality of these data throughout the reference period of the study to ensure quality and consistency. The data represent total monthly blood transfusions given by ward. Transfusions are measured as the number of transfusions completed per patient, and not as a measure of the units of blood transfused. Blood transfusion data specifically for paediatric malaria admissions were obtained from an electronic database of all paediatric malaria admissions from 2003–2008. The analyses used the data on total paediatric blood transfusions rather than paediatric blood transfusions for severe malarial anaemia from these patient records. These latter data were only available for 2003–2008, limiting the number of time points prior to malaria control scale-up. The statistical correlation between total paediatric blood transfusions and those used for severe malarial anaemia is high (0.94), justifying the use of total paediatric blood transfusion as a proxy for those used for severe malarial anaemia.

Given differing data sources and data management approaches among sources, the data on total hospital admissions and outpatient visits use different age thresholds than the malaria admission and blood transfusion data. The HMIS data report data for children under five years of age, while the electronic database on paediatric inpatient malaria admissions and blood transfusion reports data on children five years and under. Although there is a discrepancy in the age categorization across sources, this does not affect the main analysis between malaria admissions and paediatric blood transfusions since the same age threshold is used. Hereafter, for simplicity, all patient data will be referred to as paediatric cases.

For this study, paediatric admissions with severe malarial anaemia and cerebral malaria with severe anaemia were classified based on the first and second discharge diagnosis in the patient records. Severe malarial anaemia was diagnosed if haemoglobin (Hb) <5 g/100 ml. At MMH, the criteria for transfusion is haemoglobin less than or equal to 5 g/100 ml, consistent with the WHO guidelines for treating severe anaemia. In reality, blood transfusions may have been given to patients with Hb >5 at the discretion of the physician if the patient was thought to be unstable, had very high parasitaemia, experienced cardiac failure, or had co-existing malnutrition. Because the blood transfusion data for the paediatric malaria admissions were drawn directly from patient records, these transfusions are reflected in the data. Cerebral malaria with severe anaemia was diagnosed if a child has a positive malaria smear with coma (Blantrye Coma Score ≤2) with no other cause found for the coma, and also had Hb <5 g/dl.

### Analytical approach

The aim of this study was to assess the extent to which the use of blood transfusions in the paediatric ward is associated with the number of paediatric outpatient malaria visits, paediatric inpatient malaria admissions, and paediatric inpatient admissions for severe malarial anaemia. It then explores whether this association can be attributed to the scale-up of malaria control interventions in MMH’s catchment area, based on the timing of these interventions. The following presents the hypothesized logic model relating malaria control scale-up with use of paediatric blood transfusions: First, effective malaria control should reduce the incidence of malaria in the hospital’s catchment area. A decrease in malaria incidence across the population should reduce the number of paediatric outpatient malaria visits. More effective malaria control, through prevention, should decrease the number of malaria cases. In addition, more effective first-line treatment should decrease the number of severe malaria cases, including severe malarial anaemia. Together, the number of admissions for malaria should fall. Since a large portion of paediatric blood transfusions are used for malaria, a reduction in these admissions should reduce the use of paediatric blood transfusions overall.

Malaria control scale-up may also indirectly affect the use of blood transfusions in other patient wards. If the blood supply is limited at a health facility and there are competing uses for blood resources, a decline in blood transfusion use in one ward may result in greater blood availability for other wards. If blood transfusion use in the paediatric ward declines with malaria control scale-up, then more blood may be available for use in other wards. These potential spillover effects (or indirect benefits) are most likely to occur if the total blood supply is fixed or limited, and if it is rationed between wards; otherwise, such spillover effects may not exist, or may be relatively small.

All analyses used an autoregressive-moving-average (ARMA) model to account for potential autocorrelation of time-series data. By accounting for the potential correlation over time in the outcome variable, this model prevents the observed effects from being confounded by secular trends over time, which also affect the outcome. The model also accounts for potential correlation of the residual (unobserved) term over time. Based on the autocorrelation function for the monthly paediatric blood transfusion data, the best-suited model for the main analyses included two lag variables for both the autoregressive model and the moving-average model (defined as a AR(2)MA(2) model which means that there exist correlations across the two most recent time periods). The ARMA model uses a maximum likelihood estimator. To account for potential unequal variance in the error term by year (heteroskedasticity), the model used robust standard errors. All analyses were conducted using Stata SE (version 11.0, StataCorp).

The main outcome of interest was the number of monthly paediatric blood transfusions. The first analyses quantified the relationship between the use of paediatric blood transfusions and the number of paediatric malaria outpatient visits. In this model, the regression included an interaction term that identifies the 2004–2008 period which represents the time period of malaria control scale-up in MMH’s catchment area. The interaction term shows whether the relationship between paediatric malaria visits and paediatric blood transfusions changes when malaria control is scaled-up. Similar analyses were conducted using paediatric malaria admissions and paediatric admissions for severe malarial anaemia, respectively, as the independent variables.

The next set of analyses used an interrupted time series model to investigate whether the rate of use of paediatric blood transfusions is different when malaria control is scale-up and whether the average number of paediatric blood transfusions used differs as well. In these models, the independent variables included: 1) a monthly trend variable for the 2000–2003 period, 2) a variable capturing the average difference in quantity of paediatric blood transfusions used per month during the 2004–2008 period relative to the 2000–2003 period, and 3) a monthly trend variable for the 2004–2008 period. The 2000–2003 monthly trend variable represents the rate of paediatric blood transfusion use prior to malaria control scale-up in the hospital’s catchment area. The 2004–2008 monthly trend variable identifies the rate of paediatric blood transfusion use from 2004–2008, representing the malaria control scale-up period. A comparison of these two variables shows whether the rate of use is different before and after malaria control scale-up. The difference in monthly use variable identifies whether average number of monthly paediatric blood transfusions used during the 2004–2008 period is different than the monthly average used in the 2000–2003 period. A similar model was run separating the malaria control scale-up period into two periods: 2004–2006 and 2007–2008. This specification allows the monthly trend (change in use over time) and average difference in monthly use during both post scale-up periods to be different from each other, since different malaria control activities were taking place in these two periods. In addition, 2006 was a year when malaria control was limited because of the ACT shortage. By separating these years, this allows the magnitude of the effects to differ.

The model assumed that all years prior to 2004 were considered years when malaria control was not scaled up in MMH’s catchment area. The next set of analyses used the same model but included, as the dependent variable in separate regressions, the number of paediatric malaria outpatient visits, paediatric malaria admissions, and paediatric admissions for severe malarial anaemia, respectively. These analyses served to confirm whether the changes in the use of paediatric blood transfusions as a function of malaria control scale-up could be explained by the changes in malaria outpatient visits and malaria inpatient admissions related to malaria control scale-up.

Exploratory analyses were conducted to identify the relationship between paediatric blood transfusion use and total use of blood transfusions at the hospital. Similar analyses were also done using, as dependent variables, blood transfusion use in different patient wards, including the maternity, women’s, men’s, and tuberculosis ward. These analyses include an interaction term, to assess whether the relationship between blood use in the paediatric ward and blood use in other wards is different during the 2004–2008 malaria control scale-up period.

## Results

### Trends over time in paediatric malaria admissions and outpatient visits and blood transfusions at Macha Mission Hospital

Summary statistics for both paediatric outpatient malaria visits and paediatric malaria admissions at MMH show substantial declines over time (Table 1). During the period 2003–2008, paediatric malaria admissions peaked in 2003 (1,687 admissions) then followed a downward trend, with 233 paediatric malaria admissions by 2008. Despite the general decline in paediatric malaria admissions over this time period, there was an increase from 2005 to 2006 likely associated with the end of the 2004/2005 drought and the ACT drug shortage. Similar downward trends over this time period occurred for paediatric malaria outpatient visits, with an increase occurring in 2006 relative to 2005 despite a smaller relative number of total outpatient paediatric visits. Outpatient paediatric malaria visits then continued to decrease in 2007 and 2008. Malaria hospital admission data for Southern Province show similar trends with a peak in malaria admissions in 2003, followed by a subsequent decline in 2004 and 2005. Malaria admissions increase in 2006, relative to 2005, then continue to fall in 2007 and 2008. The national trend over this time period is a consistently downward trend, with admissions slightly higher in 2006 compared to 2005 [33].

**Table 1:** **Total paediatric outpatient malaria visits and inpatient malaria admissions**

Year	Outpatient malaria visits	Inpatient malaria admissions	Inpatient admissions for severe malarial anaemia
2000	6328	NA	NA
2001	5269	NA	NA
2002	4536	NA	NA
2003	5611	1687	282
2004	1660	579	89
2005	700	208	25
2006	1042	618	119
2007	969	471	74
2008	120	233	18

Note: Data for inpatient malaria admissions were only available for 2003-2008.

**Figure 2:**
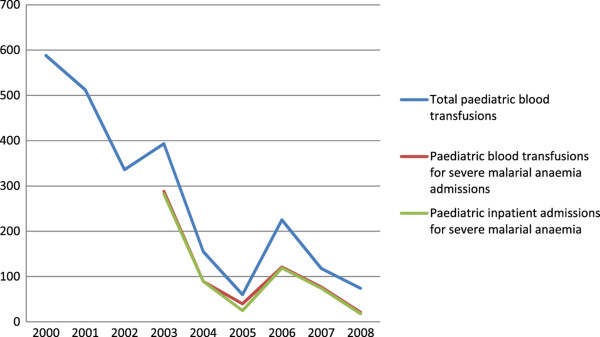
**Pediatric admissions for severe malarial anaemia and paediatric blood transfusions at Macha Mission Hospital.** Graphs shows the yearly trends in paediatric admissions for severe malarial anaemia and paediatric blood transfusions.

Over the same period, yearly paediatric blood transfusions followed similar trends as paediatric malaria admissions and outpatient visits. In 2000, there were 588 blood transfusions in the paediatric ward (Table 2). Yearly paediatric blood transfusions had fallen to 60 by 2005; they subsequently increased in 2006 to 225, and then fell to 74 transfusions in 2008. Paediatric blood transfusions for severe malarial anaemia are only available for the period 2003–2008. During this period, they followed a similar trend as total paediatric blood transfusions, decreasing from 288 in 2003 to 40 by 2005, increasing to 121 in 2006 then falling to 21 in 2008. Paediatric blood transfusions for severe malaria made up 73% of all paediatric blood transfusions in 2003. Through 2007, the proportion of all paediatric blood transfusions that were used for severe malarial anaemia admissions is 67%, on average. In 2008, this proportion fell to 28%. The yearly trend in total paediatric blood transfusions and those used for paediatric admissions for severe malaria anaemia follow a consistent trend over time; paediatric admissions for severe malaria anaemia follow exactly the same trend as the use of blood transfusions for these admissions (Figure 2).

**Table 2:** **Total pediatric blood transfusions over time (2000–2008)**

Year	Total paediatric blood transfusions	Paediatric blood transfusions for malaria admissions (percentage of total paediatric blood transfusions)	Paediatric blood transfusions for severe malarial anaemia admissions (percentage of total paediatric blood transfusions)	Paediatric blood transfusions (as percentage of all blood transfusions at hospital)
2000	588	NA	NA	73%
2001	512	NA	NA	66%
2002	336	NA	NA	57%
2003	393	288 (73%)	288 (73%)	56%
2004	155	97 (63%)	89 (57%)	31%
2005	60	42 (70%)	40 (67%)	20%
2006	225	135 (60%)	121 (54%)	41%
2007	118	81 (69%)	77 (65%)	24%
2008	74	33 (45%)	21 (28%)	22%

Note: Data for inpatient malaria admissions were only available for 2003-2008.

**Figure 3:**
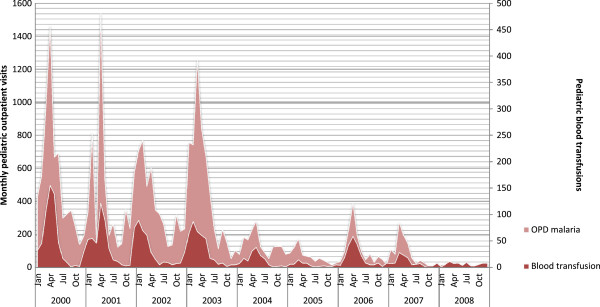
**Paediatric outpatient malaria visits and number of blood transfusions in the paediatric ward at Macha Mission Hospital.** Graph showing the similar monthly trends of paediatric blood transfusions, paediatric malaria admissions, and paediatric outpatient malaria visits.

The monthly trend for paediatric blood transfusions followed a similar trend to the monthly trend in paediatric malaria admissions and paediatric outpatient malaria visits (Figure 3). Figure 3 uses outpatient malaria visits rather than inpatient malaria admissions because these data were available over a longer time period. Outpatient malaria visits can serve as a proxy for the malaria incidence although they represent an imperfect proxy because they do not capture malaria cases which were seen at other lower level health centres or malaria cases that were treated at home. Outpatient visits also serve as a proxy for inpatient admissions since they are correlated. As the number of paediatric outpatient malaria visits decreased, the monthly number of paediatric blood transfusions also dropped substantially. The monthly paediatric blood transfusion trends also mirrored the seasonality of malaria transmission in MMH’s catchment area.

**Figure 4:**
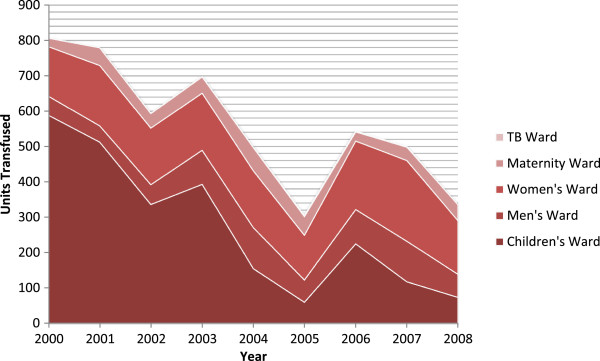
**Total number of blood transfusions by ward at Macha Mission Hospital.** Graph showing similar trends over time for the total number of blood transfusions at Macha Mission Hospital across all wards. Note: the number of blood transfusions in the tuberculosis (TB) ward is relatively small, which explains why they are not perceptible in the graph.

The trend over time for the total number of blood transfusions at MMH across all wards followed a similar trend to paediatric blood transfusions (Figure 4). During the period 2000 to 2008, the highest total number of transfusions at MMH occurred in 2000, with 807 transfusions. Total transfusions fell to 305 in 2005, then increased to 544 in 2006 and subsequently fell to 335 in 2008. In 2000, the paediatric ward accounted for 73% of total blood transfusions at MMH. The proportion fell to 20% in 2005 and 22% in 2008 (with an increase in 2006 to 41%). These data represent number of transfusions by patient ward and not units of blood transfused, which vary by patient weight. Similar trends exist for units of blood transfused.

### Association between paediatric malaria visits and admissions and paediatric blood transfusions

The main results (Table 3) show that there is both a positive correlation between paediatric malaria visits and paediatric blood transfusions as well as a positive correlation between paediatric malaria admissions (including paediatric admissions for severe malarial anaemia) and paediatric blood transfusions. In the first model (column 1), for each additional paediatric malaria outpatient visit, there were 0.07 additional paediatric blood transfusions (95% CI 0.01-0.13; *p* < 0.05), and for each additional paediatric admission for malaria, there were 0.19 additional paediatric blood transfusions (column 3, 95% CI 0.03-0.35; *p* < 0.05). Lastly, for each additional paediatric admission for severe malarial anaemia, there were 1.09 additional paediatric blood transfusions (column 5, 95% CI 0.95-1.23; *p* < 0.01). Paediatric admissions for severe malarial anaemia are a subset of all paediatric malaria admissions. This result suggests that a patient admitted for severe malarial anaemia receives one blood transfusion, with some admitted patients receiving more than one blood transfusion during their stay. It also demonstrates that the paediatric admissions for severe malarial anaemia largely explain total use of blood transfusions in the paediatric ward. The interaction terms (identifying the 2004–2008 period) are not statistically significant for paediatric malaria outpatient visits and paediatric malaria inpatient admissions (columns 2 and 4). This means that the relationship between malaria cases (outpatient visits and admissions) relative to the use of paediatric blood transfusions does not change over time, as malaria control is scaled-up. However, the interaction term is statistically significant for paediatric admissions for severe malarial anaemia (column 6). These results imply that, 2004–2008, there were 0.45 additional paediatric blood transfusions for every additional paediatric admission for severe malarial anaemia (this estimate is the linear combination of the coefficient for paediatric severe malarial admissions (1.05) and the coefficient for the interaction term (−0.60)). Yet during the 2000–2003 period there were 1.05 additional paediatric blood transfusions for every additional paediatric admission for severe malarial anaemia. These findings indicate that, during the 2000–2003 period, if there was one less paediatric admission for severe malarial anaemia, there was one less paediatric blood transfusion used, compared to a decrease of one half during the 2004–2008 period.

**Table 3:** **Association between paediatric malaria outpatient visits, malaria admissions, malaria admissions for severe malarial anaemia and paediatric blood transfusions**

	Dependent variable: paediatric blood transfusions					
	(1)	(2)	(3)	(4)	(5)	(6)
Paediatric malaria outpatient visits	0.07**	0.03**				
	(0.03)	(0.02)				
Paediatric malaria outpatient visits X period 2004-2008		0.02				
		(0.03)				
Paediatric malaria admissions			0.19**	0.21***		
			(0.08)	(0.02)		
Paediatric malaria admissions X period 2004-2008				−0.08		
				(0.06)		
Paediatric severe malarial anaemia admissions					1.09***	1.05***
					(0.07)	(0.06)
Paediatric severe malarial anaemia admissions X period 2004-2008						−0.60***
						(0.22)
Period 2004–2008 (dummy variable)		−16.59**		2.47		−0.46
		(6.78)		(4.04)		(3.01)
Constant	11.17	27.51***	3.25	1.46	2.66*	4.66
	(8.01)	(8.65)	(4.56)	(4.32)	(1.39)	(3.48)
Observations	102	102	72	72	72	72
Mean of dependent variable	23.77	23.77	14.24	14.24	14.24	14.24
Wald Chi-Squared	219.92	223.69	318.27	601.49	1.40e + 12	3483.32

*Significant at 10%; **significant at 5%; ***significant at 1%. Coefficient standard errors are noted in parentheses.

This table presents six different regressions where, in each regression, the dependent variable is the number of paediatric blood transfusions. Regressions use an autoregressive-moving-average model with 2 lags and robust standard errors. We control for the month of the year in all regressions to account for seasonal correlations. Period 2004–2008 variable is a dummy variable that takes on a value of 1 for the years 2004–2008, and 0 otherwise. The interaction term, Paediatric malaria outpatient visits X period 2004–2008, is included to identify whether the association between paediatric malaria outpatient visits and paediatric blood transfusions is different during the 2004–2008 period compared to the 2000–2003 period. The same definitions apply for the other interaction terms.

### Association between malaria control scale-up and paediatric blood transfusions

The next analyses (Table 4) show that average rate of use of paediatric blood transfusion during the 2000–2003 period is decreasing by 0.60 fewer blood transfusions per month (column 1, 95% CI .058-0.62; *p* < 0.01). During the 2004–2008 period, the rate of use remained constant over time since the variable representing the monthly trend for the 2004–2008 period is not statistically significant. However, the difference in monthly use during the 2004–2008 period is statistically significant. During the 2004–2008 period, there were, on average, 10.6 fewer paediatric blood transfusions per month compared to the 2000–2003 period (95% CI 4.01-17.15; *p* < 0.01). From an average of 38 paediatric blood transfusions during the 2000–2003 period, this represents a 28% reduction. In the second specification, the malaria control scale-up periods are separated to identify differences in effects during the 2004–2006 and 2007–2008 periods. During the 2000–2003 period, the rate of paediatric blood transfusion use decreased by 0.52 per month (column 2, 95% CI 0.32-0.72; *p* < 0.01). Average monthly use of paediatric blood transfusions during the 2004–2006 fell by 19.06, representing a 50% reduction in monthly paediatric blood use compared to 2000–2003 period. However, the rate of paediatric blood use during this period increased by 0.34 per month (column 2, 95% CI 0.14-0.54; *p* < 0.01). During the 2007–2008 period, average monthly use of paediatric blood transfusions fell by 27.5 (column 2, 95% CI 14.6-40.3; *p* < 0.01), representing a 72% reduction compared to the 2000–2003 period. The rate of blood use during the period 2007–2008 remains constant. The results in all analyses are not explained by seasonal differences in blood transfusions, because the analyses control for average monthly differences in paediatric blood transfusions, driven by factors such as malaria transmission related to rainfall.

**Table 4:** **Association between malaria control scale-up over time and paediatric blood transfusions - interrupted time series**

	Dependent variable: paediatric blood transfusions	
Independent variables	(1)	(2)
Monthly trend 2000-2003	−0.60***	−0.52***
	(0.12)	(0.10)
Difference in monthly use 2004–2008 (relative to 2000–2003)	−10.58***	
	(3.35)	
Monthly trend 2004-2008	−0.02	
	(0.04)	
Difference in monthly use 2004–2006 (relative to 2000–2003)		−19.06***
		(3.56)
Monthly trend 2004-2006		0.34***
		(0.10)
Difference in monthly use 2007–2008 (relative to 2000–2003)		−27.47***
		(6.56)
Monthly trend 2007-2008		−0.14
		(0.22)
Constant	56.59***	55.12***
	(7.62)	(7.45)
Observations	108	108
Mean of dependent variable	22.79	22.79
Wald Chi-Squared	2734.85	3740.52

*Significant at 10%; **significant at 5%; ***significant at 1%. Coefficient standard errors are noted in parentheses.

Regressions use an autoregressive-moving-average model with 2 lags and robust standard errors. We control for the month of the year in all regressions to account for seasonal correlations. For column 1, monthly trend 2000–2003 represents the rate of change by month for the outcome variable from 2000–2003. Monthly trend 2004–2008 represents the rate of change by month for the outcome variable from 2004–2008. Difference in monthly use in 2004–2008 represents the average difference in the outcome between 2000–2003 period and 2004–2008 period. For column 2, monthly trend 2000–2003 is defined as in column 1. Monthly trend 2004–2006 represents the rate of change by month for the outcome from 2004–2006. Monthly trend 2007–2008 represents the rate of change from 2007–2008. Difference in monthly use in 2004–2006 represents the average difference in outcome between 2000–2003 and 2004–2006. Difference in monthly use in 2007–2008 represents the average difference in outcome between 2007–2008 and 2000–2003.

There is evidence of seasonal effects on use of paediatric blood transfusions (Table 5). Over the study time period, on average, monthly blood transfusions were significantly lower in August through December, compared to January. Peak use occurs in April at the end of the rainy season. Kent *et al*. find that this lag occurs because the highest proportion of mosquitoes is actively transmitting at the end of the rainy season [24].

**Table 5:** **Association between malaria control scale-up over time and paediatric blood transfusions - interrupted time series**

	Dependent variable: paediatric blood transfusions	
	(1)	(2)
Monthly trend 2000-2003	−0.60***	−0.52***
	(0.12)	(0.10)
Difference in monthly use 2004–2008 (relative to 2000–2003)	−10.58***	
	(3.35)	
Monthly trend 2004-2008	−0.02	
	(0.04)	
Difference in monthly use 2004–2006 (relative to 2000–2003)		−19.06***
		(3.56)
Monthly trend 2004-2006		0.34***
		(0.10)
Difference in monthly use 2007–2008 (relative to 2000–2003)		−27.47***
		(6.56)
Monthly trend 2007-2008		−0.14
		(0.22)
February	5.79	4.88
	(4.53)	(4.70)
March	15.00	13.96
	(9.64)	(9.86)
April	26.99*	26.43*
	(14.34)	(13.81)
May	18.69	18.87
	(13.41)	(12.64)
June	−9.22	−8.40
	(11.51)	(11.79)
July	−16.37	−15.30
	(10.70)	(11.78)
August	−20.81**	−20.01*
	(10.22)	(11.76)
September	−23.94**	−23.87**
	(9.84)	(11.26)
October	−24.08***	−24.90**
	(8.96)	(9.95)
November	−24.54***	−26.06***
	(7.47)	(8.03)
December	−9.89**	−11.60**
	(4.70)	(5.56)
Constant	56.59***	55.12***
	(7.62)	(7.45)
Observations	108	108
Mean of dependent variable	22.79	22.79
Wald Chi-Squared	2734.85	3740.52

*Significant at 10%; **significant at 5%; ***significant at 1%. Coefficient standard errors are noted in parentheses.

Regressions use an autoregressive-moving-average model with 2 lags and robust standard errors. The variables are identified as in Table 3. The month variables represent a dummy variable for each month. The base (omitted) variable is January.

### Association between malaria control scale-up and paediatric malaria visits and admissions

The next results (Table 6) show that the rate of paediatric outpatient malaria visits was decreasing at a rate of 0.96 fewer outpatient malaria visits per month during the 2000–2003 period (column 1, 95% CI 0.29-1.63; *p* < 0.01). During the 2004–2008 period, the rate of paediatric outpatient malaria visits decreased at a higher rate, with 1.41 fewer malaria outpatient visits per month (95% CI 1.17-1.65; *p* < 0.01). There were also, on average, 309.4 fewer paediatric outpatient malaria visits per month during the 2004–2008 period, representing a 68% decrease compared to the 2000–2003 period. The same analyses, looking instead at paediatric malaria admissions, could not be conducted because there is only one year of pre-malaria control data, insufficient to conduct an interrupted time series analysis.

**Table 6:** **Association between malaria control scale-up and paediatric malaria visits, malaria admissions, and severe malarial admissions - interrupted time series**

	Paediatric malaria outpatient visits
Monthly trend 2000-2003	−0.96***
	(0.34)
Difference in monthly use 2004–2008 (relative to 2000–2003)	−309.38***
	(9.53)
Monthly trend 2004-2008	−1.41***
	(0.12)
Constant	527.55***
	(35.68)
Observations	102
Mean of dependent variable	258.05
Wald Chi-Squared	9.42e + 09

*Significant at 10%; **significant at 5%; ***significant at 1%. Coefficient standard errors are noted in parentheses.

Regressions use an autoregressive-moving-average model with 2 lags and robust standard errors. The monthly trend 2000–2003 represents the rate of change by month for the outcome variable from 2000–2003. Monthly trend 2004–2008 represents the rate of change by month for the outcome variable from 2004–2008. Difference in monthly use in 2004–2008 represents the average difference in the outcome between 2000–2003 period and 2004–2008 period.

The results also show that the relationship between paediatric outpatient malaria visits and inpatient malaria admissions does not change over time as malaria control is scaled-up. The analysis (Table 7) shows that, for every additional paediatric outpatient malaria visit, there were 0.3 additional paediatric malaria admissions (95% CI 0.22-0.38; *p* < 0.01). The second regression includes a control variable for the 2004–2008 period and an interaction term to identify whether this relationship changes with during this period, relative to 2000–2003 period. This relationship does not change during the 2004–2008 period as the interaction term is not statistically significant. The relationship does change though for paediatric admissions for severe malarial anaemia (column 4) since the interaction term is statistically significant. During the 2000–2003 period, for every additional paediatric malaria outpatient visit, there were 0.05 additional paediatric admissions for severe malarial anaemia (95% CI 0.03-0.07; *p* < 0.01). During the 2004–2008 period, there were 0.11 additional pediatric admissions for severe malarial anaemia; this result is the linear combination of the coefficient for outpatient malaria visits (0.05) plus the coefficient with the interaction term (0.06). This results means that the number of severe malarial admissions did not fall at the same rate as the number of outpatient malaria visits, even though both of these fell substantially during the post-malaria control scale-up period. During the post-malaria control scale-up period, the proportion of admissions for severe malarial anaemia relative to outpatient malaria visits is 11% compared to 5% during the pre-malaria control scale-up period.

**Table 7:** **Association between paediatric outpatient malaria visits and paediatric malaria admissions**

	Dependent variable: paediatric malaria admissions		Dependent variable: paediatric admissions for severe malarial anaemia	
	(1)	(2)	(3)	(4)
Paediatric outpatient malaria visits	0.30***	0.31***	0.05***	0.05***
	(0.04)	(0.06)	(0.01)	(0.01)
Paediatric outpatient malaria visits X period 2004-2008		0.13		0.06**
		(0.11)		(0.03)
Period 2004–2008 (dummy variable)		−11.80		−3.10
		(27.09)		(7.81)
Constant	5.77	9.28	2.43	2.14
	(8.29)	(24.67)	(3.06)	(8.29)
Observations	66	66	66	66
Mean of dependent variable	56.00	56.00	9.14	9.14
Wald Chi-Squared	1.31e + 11	1.38e + 12	161.61	594.00

*Significant at 10%; **significant at 5%; ***significant at 1%. Coefficient standard errors are noted in parentheses.

Regressions use an autoregressive-moving-average model with 2 lags and robust standard errors. We control for the month of the year in all regressions to account for seasonal correlations. Period 2004–2008 variable is a dummy variable that takes on a value of 1 for the years 2004–2008, and 0 otherwise.

### Association between paediatric blood transfusions and blood transfusions in other wards

As part of the exploratory analyses, the last results (Table 8) show that for every additional paediatric blood transfusion, total blood transfusions used at the hospital increased by 1.05 (column 1, 95% CI 0.95-1.15; *p* < 0.01). For all regressions, a second regression is run which includes a control variable for the 2004–2008 period and an interaction term to identify whether this relationship changes with during this period, relative to 2000–2003 period. For total blood transfusions, the relationship does not change during the 2004–2008 period compared to the 2000–2003. These findings show that there is a one-to-one relationship between the use of paediatric blood transfusions and the overall use of blood transfusions at MMH. The relationships between paediatric blood transfusions and the use of blood transfusions in other patient wards are not statistically significant.

**Table 8:** **Association between paediatric blood transfusions and blood transfusions in other wards**

	Dependent variable: total blood transfusions		Dependent variable: blood transfusions in maternity ward		Dependent variable: blood transfusions in men's ward		Dependent variable: blood transfusions in women's ward		Dependent variable: blood transfusions in TB ward	
	(1)	(2)	(3)	(4)	(5)	(6)	(7)	(8)	(9)	(10)
Paediatric blood transfusions	1.05***	1.06***	−0.00	0.00	0.02	0.03	0.01	0.04	0.00	0.00**
	(0.05)	(0.04)	(0.01)	(0.01)	(0.02)	(0.02)	(0.02)	(0.02)	(0.00)	(0.00)
Paediatric blood transfusions X period 2004-2008		0.04		0.00		−0.00		0.03		−0.00
		(0.10)		(0.03)		(0.08)		(0.07)		(0.00)
Period 2004–2008 (dummy variable)		4.47		0.45		2.98*		1.98		0.18**
		(3.28)		(0.74)		(1.78)		(1.89)		(0.09)
Constant	21.54***	18.61***	5.04***	4.65***	5.42***	3.46**	12.11***	10.25***	0.06	−0.05
	(2.43)	(2.92)	(1.16)	(1.21)	(1.32)	(1.63)	(1.17)	(1.78)	(0.11)	(0.11)
Observations	108	108	108	108	108	108	108	108	108	108
Mean of dependent variable	46.85	46.85	3.60	3.60	6.55	6.55	13.81	13.81	0.11	0.11
Wald Chi-Squared	2145.86	2427.16	2.33e + 12	1.22e + 13	314.94	238.78	12907.44	14233.20	8.56e + 10	1.05e + 07

*Significant at 10%; **significant at 5%; ***significant at 1%. Coefficient standard errors are noted in parentheses.

Regressions use an autoregressive-moving-average model with 2 lags and robust standard errors. We control for the month of the year in all regressions to account for seasonal correlations. Period 2004–2008 variable is a dummy variable that takes on a value of 1 for the years 2004–2008, and 0 otherwise.

## Discussion

### Summary of main findings

In this study, the results show that the number of paediatric admissions for severe malarial anaemia largely explain the number of paediatric blood transfusions used. This finding highlights that reducing paediatric admissions for severe malarial anaemia lowers the use of paediatric blood transfusions. The results show a one-to-one relationship between paediatric admissions for severe malarial anaemia and paediatric blood transfusion use during the 2000–2003 period. This relationship appears to be affected by malaria control scale-up. As paediatric admissions for severe malarial anaemia decrease during the 2004–2008 period, paediatric blood transfusions continue to decrease but at a slower rate. This result is not due to an increase in use of blood transfusions among these admissions during the 2004–2008 period. Actually, during the 2004–2008 period 86% of these admissions received a blood transfusions compared to 96% during the 2000–2003 period. One possible explanation is that blood transfusions for severe malarial anaemia start to make up a smaller share of all paediatric blood transfusions (28% by 2008). Some of the paediatric blood transfusions are being used for other malaria admissions, specifically malarial anaemia admissions suggesting that a higher threshold may have been used for transfusing. Some paediatric blood transfusions are also for non-malaria patients (55%). The use of paediatric blood transfusions for non-malaria patients follows a similar pattern to the use by malaria patients. The trend in paediatric blood transfusions for non-malaria admissions also follows a similar pattern over time consistent with malaria control scale-up. One probable explanation is that these non-malaria patients may have been treated successfully for malaria at another facility, but were referred to MMH because of severe anaemia as a result of the recent malaria infection. Such patients would have received a blood transfusion if their haemoglobin was less than 5 but the discharge diagnosis would not identify them as malaria patients.

The results show that fewer blood transfusions were administered on the paediatric ward in years when malaria control was scaled up in MMH’s catchment area compared to years when malaria control was not scaled up. Compared to the 2000–2003 period when malaria control was relatively limited, there was a 50% reduction in the average monthly use of paediatric blood transfusions from 2004–2006. During this period, ACT was introduced as the first line of treatment and the test-and-treat campaign was implemented (2004 and 2005). Although the monthly number of paediatric blood transfusions is lower in this period compared to 2000–2003, the rate of use was increasing during this period. One of the reasons for the increase in the rate of paediatric blood transfusions during this period is likely due to the countrywide ACT shortage in 2006. This ACT shortage may have led patients to either bypass the health centre or receive hospital referrals because of treatment shortages at the lower levels of care [28]. In addition, the shortages caused recourse to less effective treatment, primarily SP, to which resistance was prevalent, potentially leading to ineffective treatment that resulted in progression of malaria and subsequent hospitalization [34].

During the period 2007–2008, there was a 72% reduction in the monthly use of paediatric blood transfusions compared to 2000–2003. The ITN distribution campaign was rolled out during this period. The results comparing the 2004–2008 period with the 2000–2003 period are consistent with these findings, but the magnitude of the relationship with paediatric blood transfusions is smaller. This is again likely due to the increase in malaria visits and admissions in 2006 associated with the ACT shortage, which may have dampened the overall relationship. While there may have been some malaria control occurring in 2006 (through ITN distribution), the scale was not equivalent to the widespread distribution of ITNs beginning in 2007. For these reasons, the results for the two separate time periods are used to allow for the effect from these different years to differ. The decrease in the rate of paediatric blood transfusion use during the 2000–2003 suggests that there may have been limited malaria control during this period although much less pronounced than during the scale-up period from 2004 onwards.

The substantial decline in paediatric outpatient malaria visits during the 2004–2008 period is consistent with the scale-up of malaria control interventions. This time period is associated with a 68% decrease in paediatric outpatient malaria visits. Although there are no available data on malaria incidence in the hospital’s catchment area, data on outpatient visits were used as a close proxy for malaria incidence. Certain limitations exist in interpreting these data relative to malaria incidence: They do not represent confirmed cases of malaria until RDTs were introduced, and they only include visits to MMH’s outpatient department and not visits to other lower level health centres. Nonetheless, the consistent association between the timing of malaria control scale-up and the decrease in paediatric outpatient malaria visits substantiates the hypothesized pathway through which malaria control scale-up could affect paediatric blood transfusion use. In addition, the analyses show a consistent correlation over time between malaria outpatient visits and malaria inpatient admissions. There is evidence that the rate at which paediatric outpatient malaria visits decreased is greater than the rate of decrease in paediatric malaria admissions. With effective malaria control, the number of outpatient malaria visits may fall faster than admissions because the proportion of cases that present at the health facility are in greater need of admission. Another possible explanation is related to the introduction of RDTs in 2007 at MMH, since prior diagnosis based on clinical presentation has been shown to over-estimate the number of malaria cases [24]. The relative difference is small since both malaria outpatient visits and malaria admissions for severe malarial anaemia fall substantially over this time period.

There is also evidence of the seasonal use of paediatric blood transfusions, consistent with the timing of the rainy season. Fewer paediatric blood transfusions are used during dry season months relative to rainy season months, which is consistent with the low and high malaria transmission months. Overall, these findings suggest that the use of paediatric blood transfusions is driven both by differences in malaria transmission resulting from seasonal variation (including severe drought), and by differences in malaria incidence over time resulting from the scale-up of malaria control efforts.

In the context of this particular hospital, there are no identified spillover effects (or indirect benefits) from the use of paediatric blood transfusions on the use of blood transfusions in other patient wards. The evidence demonstrates that there is a one-to-one relationship between paediatric blood transfusions and total use of blood transfusions at MMH. This positive correlation suggests that there is not a fixed supply of blood at MMH and/or that the need for blood does not exceed the available supply at this particular facility. Blood does not appear to be rationed between patient wards; this explains why there are no indirect benefits on blood use in other patient wards despite the substantial differences over time in blood use in the paediatric ward. However, it is not possible to completely rule out that there could have been some rationing if paediatric patients were prioritized. The data represent blood transfusions used and not blood transfusions ordered, so it is not feasible to measure the demand or need for blood transfusions during this period. Another reason that there may be no effect is due to the measurement of blood transfusions; these are measured relative to patients and adult patients will require a greater quantity of blood per transfusion relative to paediatric patients. As a result, a large effect on paediatric blood use will translate to a smaller effect for adult patients.

### Limitations

The analytical methods in this study are used to demonstrate correlations between the variables of interest, but do not show whether the relationship is causal. One of the main limitations of the study was the absence of district level malaria control coverage data in the study area, preventing quantification of the relationship between particular malaria control interventions (ACT availability and ITN distribution) and the use of paediatric blood transfusions. Another limitation is the absence of malaria incidence data for the specific catchment area, which would strengthen the identification of the relationship between malaria control scale-up and the use of paediatric blood transfusions. Follow-up research with more detailed information about malaria control coverage and malaria incidence could more precisely quantify the relationship between malaria control scale-up and blood transfusion use.

There are certain potential confounders in the analysis that could not be controlled for. For example, there may have been changes over time in provider admitting and transfusing behavior or changes in the general quality of care at the hospital. However, changes in these factors would need to be contemporaneous with the malaria control efforts to confound the results. In general, it is unlikely that changes in provider transfusing behavior explain these results because the guidelines for transfusions did not change over this time period at MMH and were consistent with WHO requirements. Nonetheless, there may have been variations by physician in terms of blood transfusion decision-making. The threshold for transfusing a paediatric patient is haemoglobin below 5; however, a higher threshold may have been used by some physicians and this would explain why more admissions for malarial anaemia received blood transfusions during the 2004–2008 period. There may also have been changes in care-seeking behavior on the patient side with increased awareness about malaria as malaria control was scaled-up. There is no available evidence to assess this potential confounder in this context.

The main results are not confounded by seasonal variation in rainfall since the analysis includes control variables for each month. In addition, the ARMA model controls for trends over time in the outcome variable which means that it accounts for average differences such as weather patterns. However, the results may be confounded by the drought that occurred during the 2005 rainy season. Given that this drought coincided with the introduction of ACT in that year, it is not possible to distinguish, for that particular year, between the differential effect of the malaria control interventions relative to the seasonal effects from low rainfall. Nonetheless, both factors would affect malaria admissions in a similar direction.

Another potential confounder relates to the changes in blood collection processes at MMH during the study period. Until 2006, MMH managed its own blood collection through blood drives. Starting in 2006, Ministry of Health policy change disallowed local blood collection throughout Zambia, requiring MMH to obtain blood centrally. Anecdotal evidence indicates that this shift did affect blood availability and use and may have resulted in increased blood rationing within the facility. By collecting its own blood collection process prior to 2006, MMH could potentially better manage changes in the blood supply with more immediacy (such as requesting immediate replacement blood donations from family members). A large portion of the data for this study come from the period before the policy change, which suggests that the hospital could better manage blood supply needs across wards when it had its own blood banking. This would also explain the absence of any spillover effects on other patient wards. The lower number of paediatric blood transfusions post-2006 cannot be explained solely by blood supply constraints given that the proportion of blood transfusions used in the paediatric ward relative to other wards also fell, from 73% in 2000 to 22% in 2008.

The results of this study are specific to MMH, which may serve a different patient population than a semi-urban or urban hospital or a lower-tiered facility. MMH is also located in an area where malaria has typically been hyperendemic, so the seasonal effects on paediatric blood transfusions may be different than in areas where malaria is holoendemic, mesoendemic or hypoendemic. Finally, the results reflect the relationship between the particular malaria control interventions that were implemented in MMH’s catchment area. The magnitude of the effect of malaria control interventions will depend on the type of intervention and its effectiveness. Nonetheless, the trend in malaria admissions at MMH are similar to the trends in malaria admissions in Southern Province.

## Conclusions

The efficacy of blood transfusions to save the lives of children with severe malarial anaemia is well understood, and data from several studies show that the majority of paediatric blood transfusions in malaria-endemic countries in SSA are given for this reason. This study provides important insights into the relationship between paediatric malaria visits and admissions and blood transfusion use and how differences in paediatric blood transfusion use are related to the scale-up of malaria control interventions over time.

This study demonstrates the extent to which paediatric blood use is lowered as paediatric outpatient malaria visits and paediatric admissions for severe malarial anaemia decrease. It also demonstrates that the significant reduction in paediatric blood transfusion use over time is consistent with the scale-up of malaria control interventions. These interventions appear to decrease the number of malaria visits, an imperfect proxy for malaria incidence. This study is the first to investigate potential spillover effects, or indirect benefits, from reduced blood transfusion use in the paediatric ward on blood use in other patient wards. The results show that, in this particular facility, the blood supply does not appear to be fixed and there does not appear to be substantial effects from differences in blood use in the pediatric ward on blood use in other patient wards. The study paves the way for follow-up research in other facility settings to investigate the potential benefits on health outcomes for non-malaria patients at the facility level, particularly in the context of fixed or limited blood supply. Such studies are particularly relevant in the current context of critical blood supply shortages in many developing countries, where blood supply may not meet clinical demand both for malaria and non-malaria patients.

### Ethical review

This study was exempted from IRB review by the Abt Associates Institutional Review Board. The study received ethical approval from Eres Converge in Lusaka, Zambia and from the Macha Research Trust IRB. The study was also reviewed by the CDC IRB.

## Abbreviations

AL: Artemether-lumefantrine
ACT: Artemisinin-based combination therapy
AP: Atovaquone-proguanil
ARMA: Autoregressive-moving-average
Hb: Haemoglobin
HMIS: Health Management Information System
IRS: Indoor residual spraying
ITN: Insecticide-treated net
IPTp: Intermittent preventive therapy for pregnant women
MMH: Macha Mission Hospital
MIAM: Malaria Institute at Macha
OLS: Ordinary least squares
RDT: Rapid diagnostic test
SSA: Sub-Saharan Africa
SP: Sulphadoxine-pyrimethamine
WHO: World Health Organization
USAID: US Agency for International Development.
